# Factors associated with death from dengue and chikungunya virus infection during an epidemic period in Northeast Brazil: A retrospective cohort study

**DOI:** 10.1590/0037-8682-0030-2023

**Published:** 2023-06-02

**Authors:** Marcela Franklin Salvador de Mendonça, Amanda Priscila de Santana Cabral Silva, Heloísa Ramos Lacerda

**Affiliations:** 1 Universidade Federal de Pernambuco, Programa de Pós-Graduação em Medicina Tropical, Recife, PE, Brasil.; 2 Universidade Federal de Pernambuco, Centro Acadêmico de Vitória, Centro de Saúde Coletiva, Vitória de Santo Antão, PE, Brasil.

**Keywords:** Dengue, Chikungunya fever, Death, Epidemiology, Survival

## Abstract

**Background:**

We investigated the time to death and factors associated with deaths from dengue and chikungunya during the first epidemic after the introduction of the chikungunya virus in Northeastern Brazil.

**Methods:**

This retrospective cohort study was conducted in Pernambuco between 2015 and 2018. Logistic regression was used to identify independent risk factors. The probability of survival among individuals with different arbovirus infections was estimated and the survival curves were compared using log-rank tests.

**Results:**

The lethality coefficients for dengue and chikungunya viruses were 0.08% and 0.35%, respectively. The chance of death due to chikungunya infection increased progressively from the age of 40 years. At 40-49 years, the odds ratio was 13.83 (95%CI, 1.80-106.41). At 50-59 years and 60 years or older, the odds ratio was 27.63 (95%CI, 3.70-206.48); and 78.72 (95%CI, 10.93-566.90), respectively. The probability of death associated with dengue virus infection increased from the age of 50 years. Among patients aged 50-59 years and 60 years or older, the odds ratio was 4.30 (95%CI, 1.80-10.30) and 8.97 (95%CI, 4.00-20.0), respectively. Independent factors associated death were headache and age of 50 years or older for dengue; and headache, nausea, back pain, intense arthralgia, age 0-9 years or 40 years and older, and male sex for chikungunya. The ratio between mortality rates revealed that the time to death from dengue was 2.1 times faster than that from chikungunya (95%CI, 1.57-2.72).

**Conclusions:**

The time to death was shorter in patients with dengue than in those with chikungunya disease. This study reinforces the need for faster and more effective decision-making in public health services to enhance patient outcomes and minimize mortality.

## INTRODUCTION

Arboviruses are a growing public health problem worldwide, mainly because of their potential to cause extensive epidemics, affecting numerous individuals and resulting in severe morbidities including neurological, joint, and hemorrhagic manifestations^1^. Mortality due to chikungunya has been lower than that of a typical epidemic^2^. Between 2002 and 2014, there were dengue epidemics with numerous severe cases that resulted in death in children and older adults^3^.

During an epidemic, the rapid transmission of chikungunya virus (CHIKV) can overload surveillance services, resulting in difficulties in analyzing and identifying risk factors for death among individuals with chikungunya^4^. The simultaneous circulation of dengue virus (DENV) and CHIKV in Brazil makes it more difficult to characterize deaths related to these arboviruses. The relative contribution of dengue and chikungunya to mortality is unknown. Moreover, identification of more precise characteristics to differentiate between dengue and chikungunya is warranted.

Factors associated with the risk of severe chikungunya include nephritis, cardiac arrhythmias, diabetes, and advanced age^5^ ^,^ ^6^. People with advanced age or chronic diseases have a higher risk of developing severe dengue and a higher risk of death^3^. However, there is no consensus regarding which factors are associated with the highest risk of death from these arboviruses. The lack of characteristic manifestations of these two arboviruses also contributes to difficulties in determining the cause of death, especially in the absence of an epidemic when the mortality rate is low.

In 2017, the Northeast region of Brazil had the highest number of dengue (84.051 cases; 35,2%) and chikungunya (141.363 cases; 76,6%) cases relative to the total number of cases nationwide (239,076 dengue cases and 184,458 chikungunya cases)^7^. Although all federal units register autochthonous transmission of CHIKV, the incidence and mortality are concentrated in the states of Northeast Brazil^8^.

In 2015, Ceará was the most affected state in Northeastern Brazil (62 deaths), and Pernambuco was the state with the second highest number of confirmed dengue deaths (20 cases)^9^. Among fatal cases, a positive laboratory diagnosis, immunohistochemistry for the antigen, detection of serum immunoglobulin M (IgM) antibodies using an enzyme-linked immunosorbent assay (ELISA), and virus detection through reverse-transcription polymerase chain reaction (RT-PCR) for any of the arboviruses cannot definitively confirm the specific arbovirus responsible for death^10^. Therefore, a thorough investigation of other factors that may be involved in DENV or CHIKV infection is necessary. The objective of this study was to investigate the factors associated with death from dengue and chikungunya during the first chikungunya epidemic after the introduction of CHIKV to the state of Pernambuco, Northeastern Brazil. Our results may contribute to the characterization of risk factors for death, describe the time to death in dengue and chikungunya, provide evidence for the biological and clinical characteristics associated with death, and contribute to the development of new strategies for the prevention and control of arbovirus-related deaths.

## METHODS

### Study design and location

A retrospective cohort study was conducted from 2015 to 2018 to investigate the factors associated with deaths from dengue and chikungunya during the first chikungunya epidemic after the introduction of CHIKV to the state of Pernambuco. Pernambuco is in the Northeastern region of Brazil, with an area of 98,146,315 km², including 184 municipalities and the state district of the island of Fernando de Noronha. In 2021, the estimated population was 9,674,793 and the population density was 89.62 inhabitants/km² ^11^ ^,^ ^12^. There are 12 health authorities distributed across four state macro-regions: Metropolitan: I, II, III, XII; Agreste: IV and V; Hinterland: VI, X, XI; Vale do São Francisco and Araripe: VII, VIII, and IX. These regions comprise geographically contiguous municipalities with similar cultural, economic, and social identities, and shared communication and transport networks^12^.

### Study population

The study population consisted of all confirmed cases of dengue and chikungunya in the state of Pernambuco registered in the Notifiable Diseases Information System (SINAN) and the Mortality Information System (SIM) of the Executive Secretariat of Health Surveillance in the State of Pernambuco Health Department from 2015 to 2018. Initially, a search was conducted for deaths due to dengue and chikungunya in the SINAN and SIM databases. Those who met the inclusion criteria were included in the study. Subsequently, cases were obtained only from SINAN, due to the overestimation of deaths using SIM. Confirming or disregarding a death registered in SINAN requires a comprehensive home and hospital investigation, and is dependent on complementary information regarding the epidemiological and clinical aspects of the patient. Standardized instruments were used for the investigation, namely the Arbovirus Death Investigation Report - Medical Records (Hospital) and the Arbovirus Death Investigation Report - Interview (Home), both of which were used by the Arbovirus Surveillance Management of the Executive Secretariat of Health Surveillance at the State of the Pernambuco Health Department. These data supported case discussions by the State Discussion Committee for Deaths by Dengue and Other Arboviruses, enabling the committee to confirm or disregard deaths reported in SINAN. The data were cleaned, and duplicates and cases with incomplete records were excluded.

### Inclusion and exclusion criteria

The inclusion criteria were all deaths with infection confirmed by positive visceral samples for DENV or CHIKV using RT-PCR or immunohistochemistry, IgM serum antibody detection using ELISA, or positive blood test for DENV or CHIKV using RT-PCR, and clinical and epidemiological criteria. The inclusion criteria for controls were serum IgM antibody detection using ELISA, virus detection using RT-PCR, or clinical and epidemiological criteria, with favorable outcomes (survivors). The exclusion criteria were records from home and hospital investigation reports with incomplete information or missing data in SINAN.

### Variables and statistical analysis

To define the variables, the criteria for the differential diagnosis between DENV and CHIKV infection were described in the Protocol for the Investigation of Deaths by Urban Arboviruses in Brazil (DENV, CHIKV, and Zika virus) from the Ministry of Health^13^ and characteristics reported in the literature were used^4^.

Pearson’s chi-square test was used to compare characteristics between groups. Odds ratios (OR) were calculated for age group and sex. Pearson's chi-square tests were performed using Epi Info for Windows version 7.2. Logistic regression was used to identify independent risk factors. The variables with a statistical significance level of p < 0.05 and a significant OR were retained in the final multivariable logistic regression model. The logistic regression analyses were performed using Stata (version 14.2; StataCorp, College Station, Texas, USA).

The Kaplan-Meier method was used to perform survival analysis and plot survival curves^14^ based on the time interval from onset of infection to death^15^. The log-rank test was performed to compare survival between groups, with statistical significance of p<0.001. The log-rank test is used to compare the survival curves between two or more groups^14^.Factors associated with deaths in the two groups (dengue and chikungunya) were evaluated using the chi-square test^15^. Hazard ratios (HRs) were estimated with 95% confidence intervals (CIs). The HR, which can be interpreted as the relative risk of the occurrence of an event as a function of time, was used to calculate the ratio between the dengue and chikungunya mortality rates. HRs were calculated using Cox proportional hazards regression^16^ ^,^ ^17^ ^,^ ^18^. The Kaplan-Meier and Cox regression analyses were performed using Stata (version 14.2; StataCorp, College Station, Texas, USA).

### Ethical issues

The study was conducted in accordance with the principles of the Declaration of Helsinki. Access to the raw data used in this research was authorized by the Executive Secretariat of Health Surveillance in the State of Pernambuco Health Department, and was approved by the Research Ethics Committee of the Federal University of Pernambuco (Resolution 466/2012). The Research Ethics Committee waived the requirement for informed consent owing to the use of secondary data. The data used in this study were anonymized before use.

## RESULTS

During the study period, 227 deaths due to arbovirus infections were reported, with 100 deaths from dengue and 127 deaths from chikungunya. A total of 122,970 patients with confirmed dengue and 35,758 with confirmed chikungunya survived.

For the analysis of signs, symptoms, and pre-existing diseases, 16 dengue deaths, 79,287 dengue survivors, 9 chikungunya deaths, and 19,450 chikungunya survivors were excluded because they did not have complete information. A total of 84 dengue deaths and 43,683 dengue survivors and 118 chikungunya deaths and 16,308 chikungunya survivors were included in the analysis.

### Description of deaths from dengue and chikungunya

The case fatality rates of dengue and chikungunya were 0.08% and 0.35%, respectively. Deaths predominated in men aged 60 years or older for both dengue (51%) and chikungunya (55%). Moreover, deaths predominated among non-white individuals. These deaths were predominantly laboratory confirmed as being caused by DENV or CHIKV infection. More than 80% of the deaths from dengue and chikungunya occurred among residents of the metropolitan health macro-region, covering the I, II, III, and XII health regions of the state of Pernambuco, with the majority of municipalities having populations over 500,000 (Table 1).

**TABLE 1:** Characteristics of patients who died from dengue and chikungunya.

Variable	Dengue		Chikungunya		
	n^a^	%	n^b^	%
**Age group (years)**				
0 to 9	8	8	18	14
10 to 19	7	7	3	2
20 to 29	11	11	1	1
30 to 39	7	7	4	3
40 to 49	11	11	12	9
50 to 59	18	18	19	15
60 +	38	38	70	55
**Sex**				
Male	51	51	70	55
Female	49	49	57	45
**Race/skin color** ^c^				
White	23	32	17	28
No-white	49	68	43	72
**Confirmation criterion**				
Laboratory	84	84	90	71
Epidemiological-clinical	16	16	37	29
**Health macro-region**				
Metropolitan	85	85	110	87
Agreste	14	14	12	10
Hinterland	0	0	4	3
Vale do São Francisco and Araripe	1	1	0	0
**Population**				
500,000	95	95	109	85.8
100,000-1,500,000	5	5	13	10.2
50,000-100,000	0	0	5	3.9
20,000-50,000	0	0	0	0.0
<20,000	0	0	0	0.0

^a^ Deaths from dengue = 100; ^b^ Deaths from chikungunya = 127; ^c^ Race/skin color: total for dengue = 72; total for chikungunya = 60.

### Dengue analysis

Individuals aged 60 years or older were approximately nine times more likely to die from dengue (OR, 8.97; 95%CI, 4.00-20.10) than those in the reference age group (10 to 19 years). The risk of death was also significantly elevated in the 50-59 years age group (OR, 4.30; 95%CI, 1.80-10.30). The risk of death from dengue was significantly higher in males than in females (OR, 1.57; 95%CI, 1.06-2.32). Dengue survivors were most likely to be aged 20 to 29 years (19%), and female (60%). (Table 2).

**TABLE 2:** Association between age group, sex, and death from dengue or chikungunya.

Variable	Deaths	Survivors		
	n^a^ (%)	n^b^ (%)	OR^c^	95%CI^d^
**Dengue**				
**Age group (years)**				
0 to 9	8 (8)	18,367 (15)	1.23	0.44-3.38
10 to 19	7 (7)	19,714 (16)	1.00 (ref)	-
20 to 29	11 (11)	23,106 (19)	1.34	0.52-3.46
30 to 39	7 (7)	21,473 (17)	0.92	0.32-2.62
40 to 49	11 (11)	16,599 (13)	1.87	0.72-4.81
50 to 59	18 (18)	11,782 (10)	4.30	1.80-10.30
60 +	38 (38)	11,929 (10)	8.97	4.00-20.10
**Sex** ^e^				
Male	51 (51)	48,951 (40)	1.57	1.06-2.32
Female	49 (49)	73,881 (60)	1.00 (ref)	-
**Chikungunya**				
**Age group**				
0 to 9	18 (14)	3,644 (10)	30.85	4.12-231
10 to 19	3 (2)	4,409 (12)	4.25	0.44-40.87
20 to 29	1 (1)	6,246 (17)	1.00 (ref)	-
30 to 39	4 (3)	6,191 (17)	4.03	0.45-33.12
40 to 49	12 (9)	5,419 (15)	13.83	1.80-106
50 to 59	19 (15)	4,295 (12)	27.63	3.70-206
60 +	70 (55)	5,554 (16)	78.72	10.93-567
**Sex** ^f^				
Male	70 (55)	13,044 (36)	2.13	1.50-3.03
Female	57 (45)	22,699 (63)	1.00 (ref)	-

Pearson's chi-square test. ^a^ Deaths from dengue = 100; deaths from chikungunya = 127; ^b^ Survivors from dengue = 122,970; survivors from chikungunya = 35,758; ^c^ OR = odds ratio; ^d^ 95%CI = 95% confidence interval; ^e^ Missing data: 138 Survivors; ^f^ Missing data: 15 Survivors.

The signs and symptoms significantly associated with death included headache, vomiting, diabetes, hematologic disease, chronic kidney disease, and arterial hypertension (Table 3).

**TABLE 3:** Signs, symptoms, and pre-existing diseases of patients with dengue and chikungunya according to their survival status.

Variable	Deaths	Survivors	
	n^a^ (%)	n^b^ (%)	p
**Dengue**			
Fever	72 (86)	38,918 (89)	0.321
Myalgia	48 (57)	29,002 (66)	0.073
Headache	36 (43)	30,851 (71)	<0.001
Rash	19 (23)	15,109 (35)	0.021
Vomiting	37 (44)	10,344 (24)	<0.001
Nausea	18 (21)	13,216 (30)	0.078
Back pain	9 (11)	5,227 (12)	0.724
Conjunctivitis	1 (1)	1,395 (3)	0.297
Arthritis	9 (11)	3,021 (7)	0.171
Intense arthralgia	29 (35)	13,647 (31)	0.517
Petechiae	6 (7)	3,307 (8)	0.882
Leucopenia	2 (2)	1,172 (3)	0.864
Positive tourniquet test	2 (2)	718 (2)	0.596
Retro-orbital pain	8 (10)	5,475 (13)	0.405
Diabetes	8 (10)	239 (1)	<0.001
Hematologic disease	2 (2)	124 (0)	<0.001
Liver disease	1 (1)	113 (0)	0.094
Chronic kidney disease	2 (2)	75 (0)	<0.001
Hypertension	8 (10)	543 (1)	<0.001
Dyspepsia	0 (0)	146 (0)	0.596
**Chikungunya**			
Fever	103 (87)	14,636 (90)	0.381
Myalgia	69 (58)	9,525 (58)	0.988
Headache	45 (38)	9,761 (60)	<0.001
Rash	47 (40)	5,887 (36)	0.400
Vomit	34 (29)	3,217 (20)	0.014
Nausea	10 (8)	3,626 (22)	<0.001
Back pain	14 (12)	3,277 (20)	0.026
Conjunctivitis	4 (3)	818 (5)	0.420
Arthritis	29 (25)	2,471 (15)	0.005
Intense arthralgia	73 (62)	12,242 (75)	<0.001
Petechiae	5 (4)	673 (4)	0.952
Leucopenia	3 (3)	303 (2)	0.584
Positive tourniquet test	1 (1)	98 (1)	0.730
Retro-orbital pain	6 (5)	1,162 (7)	0.390
Diabetes	13 (11)	198 (1)	<0.001
Hematologic disease	0 (0)	37 (0)	0.604
Liver disease	4 (3)	74 (0)	<0.001
Chronic kidney disease	7 (6)	40 (0)	<0.001
Hypertension	27 (23)	418 (3)	<0.001
Dyspepsia	2 (2)	56 (0)	0.014

Pearson's chi-square test. ^a^ Deaths from dengue = 84; deaths from chikungunya = 118; ^b^ Survivors from dengue = 43,683; survivors from chikungunya = 16,308.

The multivariable analysis showed that factors associated with death from dengue included headache (OR 3.60, 95%CI, 2.31-5.62), age of 50 to 59 years (OR, 2.53; 95%CI, 1.28-4.98) and 60 years or older age (OR, 5.69; 95%CI, 3.51-9.22) (Table 4).

**TABLE 4:** Factors associated with deaths among patients with dengue and chikungunya.

Variable	OR^a^	95%CI^b^	p^c^
**Dengue** ^d^			
**Signs, symptoms and pre-existing diseases**			
Headache	3.60	2.31-5.62	<0.001
**Age group (years)**			
50 to 59	2.53	1.28-4.98	0.007
60+	5.69	3.51-9.22	<0.001
**Chikungunya** ^e^			
**Signs, symptoms and pre-existing diseases**			
Headache	2.03	1.35-3.07	<0.001
Nausea	4.55	2.18-9.47	<0.001
Back pain	2.14	1.13-4.04	0.020
Intense arthralgia	2.73	1.83-4.07	<0.001
**Age group (years)**			
0 to 9	5.34	2.22-12.82	<0.001
40 to 49	3.85	1.47-10.08	0.006
50 to 59	9.70	4.19-22.50	<0.001
60+	23.54	11.11-49.86	<0.001
**Sex**			
Male	2.44	1.68-3.55	<0.001

Multivariable logistic regression. ^a^ OR = odds ratio; ^b^ 95%CI = 95% confidence interval; ^c^ p= Significant p<0.05; ^d^ Deaths from dengue = 84; survivors from dengue = 43,683; ^e^ Deaths from chikungunya = 118; survivors from chikungunya = 16,308; *Adjusted for age group and sex.

### Chikungunya analysis

Individuals aged 60 years or older were approximately 80 times more likely to die from chikungunya (OR, 78.72; 95%CI, 10.93-566.90) than those in the reference age group (20-29 years). Compared with the reference aged group, the risk of death from chikungunya was also significantly increased in the 0 to 9 years (OR, 30.85; 95%CI, 4.12-231.22), 40 to 49 years (OR, 13.83; 95%CI, 1.80-106.41), and 50 to 59 years (OR, 27.63; 95%CI, 3.70-206.48) age groups. The risk of death from chikungunya was significantly higher in males than in females (OR, 2.13; 95%CI, 1.50-3.03). Chikungunya survivors were most likely to be aged 20 and 39 years (34%) and female (63%) (Table 2).

The signs and symptoms that were significantly associated with death included headache, vomiting, nausea, back pain, arthritis, severe arthralgia, diabetes, liver disease, chronic kidney disease, hypertension, and dyspepsia (Table 3).

The independent factors associated with death from chikungunya were headache (OR, 2,03; 95%CI, 1.35-3.07), nausea (OR, 4,55; 95%CI, 2.18-9.47), back pain (OR, 2,14; 95%CI, 1.13-4.04), intense arthralgia (OR, 2,73; 95%CI, 1.83-4.07), age 0 to 9 years (OR, 5.34; 95%CI, 2.22-12.82), 40 to 49 years (OR, 3.85; 95%CI, 1.47-10.08), 50 to 59 years (OR, 9.70; 95%CI, 4.19-22.50), or 60 years or older (OR, 23.54; 95%CI, 11.11-49.86); and male sex (OR, 2.44; 95%CI, 1.68-3.55), as shown in Table 4.

### Survival analysis

**FIGURE 1:**
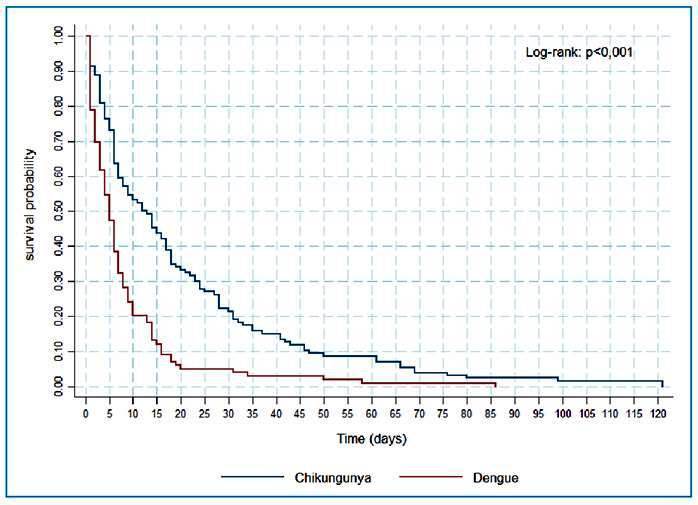
Time to death among patients who died from dengue and chikungunya.

The Kaplan-Meier survival curve indicated that the time to death was shorter among individuals who died from dengue than in those who died from chikungunya, and that the risk of death within 10 days of onset was significantly higher in those who died from dengue (log-rank: p<0.001) (Figure 1). The hazard ratio, revealed that among individuals with arbovirus infections, those with dengue were more than twice as likely to die than those with chikungunya (HR, 2.1; 95%CI, 1.57-2.72).

## DISCUSSION

This study included a large sample of individuals with arbovirus infections. The mortality rate was less than 1% for both dengue (0.08%) and chikungunya (0.35%). This result is consistent with the findings of a recent study conducted in Ceará State, Northeastern Brazil, in which the reported chikungunya mortality rate was 0.21%^4^. Our data showed that the mortality rate due to chikungunya was approximately four times higher than that due to dengue.

In CHIKV infection, mortality increased progressively from the age of 40 years, and was also elevated in children aged 0 to 9 years. In DENV infection, mortality increased from the age of 50 years. Consistent with the literature, the highest risk of death in CHIKV infection was among people over 60 years of age^2^ ^,^ ^4^ ^,^ ^19^ ^-^ ^23^. However, our results highlight a high risk of death from CHIKV infection among children and that mortality appeared to increase progressively every decade after the age of 40 years, with a peak after 60 years. We also found that the mortality risk associated with dengue was higher after 50 years of age.

Headache was an independent prognostic factor associated with death from dengue. In contrast, the independent prognostic factors associated with death from chikungunya included headache, nausea, back pain, and intense arthralgia. These data suggest that patients with chikungunya who present with headache, nausea, back pain, and intense arthralgia should receive greater attention because they are more likely to experience a fatal outcome. The risk of death from dengue was also higher in males than in females. A retrospective study conducted in Northern India showed that dyspnea, leukocytosis, and acute kidney injury were significantly associated with mortality due to dengue^24^. Although we did not analyze all these factors due to the limitations of the information collected, it is important to note that these associations may be influenced by other factors as well as by host immunity^25^. In our study, the only symptom that was independently associated with death in dengue was headache.

In this study, headache, nausea, back pain, and intense arthralgia were associated with deaths from dengue and chikungunya. This may be because people with these symptoms are more likely to seek medical care. Most patients with CHIKV infection are symptomatic^26^ and it is important to provide healthcare and treatment without delay to optimize patient outcomes and reduce the risk of death.

Among those with fatal infections, the time to death was shorter in those with dengue than in those with chikungunya, although the case fatality rate of chikungunya was higher than that of dengue. These findings, defy the conventional view that the greatest impairment in chikungunya occurs in the chronic phase, with manifestations of arthritis that can persist for months or years^27^. Although chronic inflammatory rheumatism occurs in approximately 25% of chikungunya cases and 14% to chronic arthritis occurs in 14% of chikungunya cases^28^, our findings highlight the urgent need to review clinical protocols for the early detection and management of potentially severe cases to prevent disease progression to death.

The key limitations of this study are related to its design. We used secondary data from official notification records. However, some potentially relevant information was not available. Information on previous infections with another DENV serotype was not available. Hence, we could not analyze this variable, although it may have contributed to mortality. Furthermore, surveillance services in Brazil are slow to identify deaths; consequently, data on CHIKV deaths in Brazil may have been underestimated^29^.

## CONCLUSIONS

Advanced age and male sex were associated with higher mortality rates in both dengue and chikungunya. Multivariable regression analysis, adjusted for age and sex, showed that headache was an independent prognostic factor associated with death from dengue. In contrast, headache, nausea, back pain, and intense arthralgia were signs of severity that were independent prognostic factors associated with death from chikungunya. Other independent factors associated with death from dengue were age 50 years and older, and those of deaths from chikungunya were age from 0 to 9 years or 40 years and older, and male sex. Hence, these data may be used to characterize deaths caused by DENV and CHIKV infection. Although the case fatality rate was lower in patients with dengue than in those with chikungunya, the time from onset to death was shorter in fatal cases of dengue than in fatal cases if chikungunya. To our knowledge, this study is the first to report this finding. By identifying the factors associated with death, these findings may contribute to the classification of the characteristics of infections caused by these arboviruses and the development of new strategies to prevent arbovirus infections and minimize mortality.
